# Characterization of the crystal structure, kinematics, stresses and rotations in angular granular quartz during compaction

**DOI:** 10.1107/S1600576718006957

**Published:** 2018-06-28

**Authors:** Ryan C. Hurley, Eric B. Herbold, Darren C. Pagan

**Affiliations:** a Johns Hopkins University, Baltimore, MD 21218, USA; b Cornell High Energy Synchrotron Source, Ithaca, NY 14853, USA; c Lawrence Livermore National Laboratory, Livermore, CA 94550, USA

**Keywords:** granular materials, rotations, fracture, X-ray computed tomography, grain morphology, three-dimensional X-ray diffraction

## Abstract

Three-dimensional X-ray diffraction (3DXRD), a method for quantifying the position, orientation and elastic strain of large ensembles of single crystals, has recently emerged as an important tool for studying the mechanical response of granular materials during compaction. Applications have demonstrated the utility of 3DXRD and X-ray computed tomography (XRCT) for assessing strains, particle stresses and orientations, inter-particle contacts and forces, particle fracture mechanics, and porosity evolution *in situ*. Although past studies employing 3DXRD and XRCT have elucidated the mechanics of spherical particle packings and angular particle packings with a small number of particles, there has been limited effort to date in studying angular particle packings with a large number of particles and in comparing the mechanics of these packings with those composed of a large number of spherical particles. Therefore, the focus of the present paper is on the mechanics of several hundred angular particles during compaction using *in situ* 3DXRD to study the crystal structure, kinematics, stresses and rotations of angular quartz grains. Comparisons are also made between the compaction response of angular grains and that of spherical grains, and stress-induced twinning within individual grains is discussed.

## Abstract 2

Three-dimensional X-ray diffraction and X-ray computed tomography are used to study the grain-scale response of angular granular materials to understand grain kinematics, stresses and rotations during compaction and to compare the responses of angular grains with those of spherical grains.

## 1. Introduction   

X-ray computed tomography (XRCT) has been increasingly used to characterize the *in situ* mechanical behavior of granular materials during compaction. Early work primarily used laboratory-based XRCT images with relatively coarse resolutions to examine porosity and related shear-band structures in sands under triaxial compression (Desrues *et al.*, 1996 ▸; Bésuelle *et al.*, 2000 ▸; Alshibli, Batiste *et al.*, 2000 ▸; Alshibli, Sture *et al.*, 2000 ▸). High-resolution laboratory-based XRCT was subsequently used to investigate the structure of sphere packings containing more than 100 000 grains (Aste *et al.*, 2004 ▸, 2005 ▸) and pore space for fluid flow (Turner *et al.*, 2004 ▸; Sakellariou *et al.*, 2007 ▸). Several reviews (*e.g.* Desrues *et al.*, 2010 ▸) highlight these and provide related examples of early applications.

Synchrotron-based XRCT and three-dimensional X-ray diffraction (3DXRD) have recently emerged as powerful tools for examining the grain-scale behavior of granular materials during compaction. A small number of single-crystal grains were initially studied using XRCT and 3DXRD during loading in order to understand the local and bulk stress states in the samples (Hall *et al.*, 2011 ▸; Alshibli *et al.*, 2013 ▸; Cil & Alshibli, 2014 ▸; Hall & Wright, 2015 ▸). More recently, single crystals and near-single-crystal natural sands have been studied using XRCT and 3DXRD in order to characterize contact morphology, grain kinematics, intra-grain stresses, continuum stresses, inter-particle forces, porosity, intra-grain crystal orientations and grain fracture mechanics (Alshibli *et al.*, 2013 ▸; Druckrey *et al.*, 2016 ▸; Druckrey & Alshibli, 2016 ▸; Cil *et al.*, 2017 ▸; Hurley *et al.*, 2016 ▸, 2018 ▸; Hurley, Hall & Wright 2017 ▸; Hurley, Lind *et al.*, 2017 ▸). These studies have elucidated inter-grain contact morphology (Druckrey *et al.*, 2016 ▸), structure–property relationships (Hurley, Lind *et al.*, 2017 ▸), mechanical stiffnesses at various length scales (Hurley, Hall & Wright, 2017 ▸), stress heterogeneity (Hurley *et al.*, 2016 ▸; Hurley, Hall & Wright 2017 ▸; Hurley, Lind *et al.*, 2017 ▸), response to cyclic loading (Hurley, Hall & Wright, 2017 ▸) and the stress states in grains prior to fracture (Alshibli *et al.*, 2013 ▸; Druckrey & Alshibli, 2016 ▸; Cil *et al.*, 2017 ▸; Hurley *et al.*, 2018 ▸). Related studies have also illustrated the utility of integrating these measurements with numerical simulations in order to calibrate model parameters (Imseeh & Alshibli, 2018 ▸). Although XRCT and 3DXRD have been employed in important studies of angular grains (Cil *et al.*, 2017 ▸; Imseeh & Alshibli, 2018 ▸), the majority of prior work has focused on spherical grains or a small number of angular grains. Furthermore, few studies have directly compared the mechanics of spherical and angular grain packings using these measurements.

The main goal of this paper is to investigate the compaction response of angular grains. We focus on examining intra-grain crystal structure and grain rotations, which are known to play an important role in the mechanical response of granular materials and, in the case of rotations, play an important role in micromorphic theories describing their behavior (Chen & Lan, 2009 ▸; Goddard *et al.*, 2007 ▸). We compare the responses of angular granular materials with the responses of spherical granular materials reported in prior studies because the role of grain shape in material theories and models is an important contemporary issue in granular mechanics (Kawamoto *et al.*, 2016 ▸). We also discuss stress-induced twinning in individual grains that appears to have occurred during both sample preparation and compaction. The remainder of the paper is organized as follows: §2 describes the synchrotron experiments, granular material, loading protocol, 3DXRD and intra-grain crystal structure analysis, and XRCT analyses used in this work. §3 provides a characterization of grain kinematics, stresses and rotations during compaction of the granular material. This section also discusses correlations between grain rotations and other mechanical responses, including grain displacements, grain angularity and grain stresses as well as the initial packing of the sample. §4 provides a brief discussion and concluding remarks.

## 2. Experimental   

The granular quartz used in the experiment was fabricated from a monolithic block of hydrothermally grown, electronic grade single-crystal α-quartz supplied by Sawyer Technical Materials (LLC). The block was first manually chiseled to produce fragments amenable to ball milling. Ball milling proceeded by placing a 0.5 inch (12.7 mm) diameter stainless-steel ball into a stainless-steel vial (SPEX SamplePrep mixer/mill 800D ball-mill) with the quartz fragments and milling at room temperature without any processing control agent for approximately 30 s. After ball milling, grains were subjected to vibratory sieving for approximately 5 min with multiple sieve sizes. Grains retained on a standard number 80 mesh (177 µm) and passing a standard number 60 mesh (250 µm) were selected for the experiments. This range of particle sizes was selected in order to provide a large number of grains (several hundred) in the sample volume with a sufficient size to ensure that Bragg diffraction peaks did not significantly overlap (a requirement for 3DXRD; Bernier *et al.*, 2011 ▸; Oddershede *et al.*, 2010 ▸).

**Figure 1:**
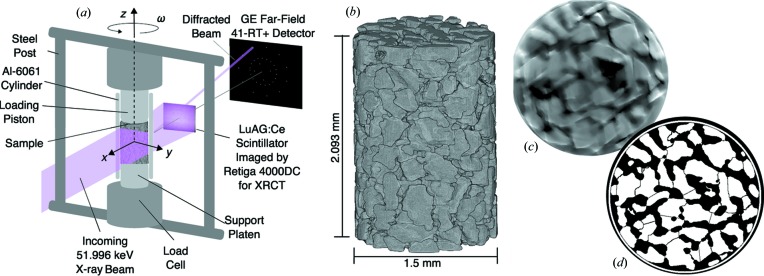
(*a*) Schematic of experimental setup and CHESS F2 hutch detectors. (*b*) XRCT reconstruction of load step 0. (*c*) Horizontal slice through XRCT reconstruction of load step 0. (*d*) Slice through segmented three-dimensional volume at the same horizontal slice as shown in (*c*).

Angular grains were poured through a funnel into a 10 mm tall, 1.5 mm diameter aluminium (Al-6061) cylinder. Al-6061 was selected as a cylinder material to provide a combination of stiffness and low X-ray absorption. Before pouring, the cylinder was placed on a stainless-steel support platen of 1.5 mm diameter that, after pouring, was inserted into the compact load frame present at the F2 beamline of the Cornell High Energy Synchrotron Source (CHESS). A schematic of the compact load frame and the near- and far-field detectors at F2 are shown in Fig. 1 ▸. The load frame features a load cell and steel support posts, and rests atop a translation and rotation stage that permits motion in the *x*, *y* and *z* directions, and rotation about the *z* axis by an angle ω. In the F2 hutch, the hardware used for transmission radiography measurements (and therefore XRCT) is a Retiga 4000DC CCD camera, with variable objective lenses, focused on an LuAG:Ce scintillator. The far-field detector is a GE 41-RT+ area detector with 2048 × 2048 pixels.

Experiments were performed by lowering the compact-load-frame loading piston into the aluminium cylinder and compressing the granular sample until a desired load level was reached. The displacement of the loading piston was then held constant while the granular sample was illuminated in 1 mm tall volumes with a 51.996 keV monochromatic parallel X-ray beam. While illuminated, the sample was rotated through two 360° rotations to make transmission radiography and diffraction measurements. During the first rotation, transmission radiography measurements were made with a 5× objective, resulting in a 1.48 µm pixel size, at angular increments of 0.25° for the first (approximately) 140° of angular rotation for which the sample was not obstructed by the compact-load-frame support posts. During the second rotation, 3DXRD measurements were made with the GE far-field area detector at angular increments of 0.25° for the ∼280° of angular rotation for which the sample was not obstructed by the support posts.

### 2.1. Loading   

**Figure 2:**
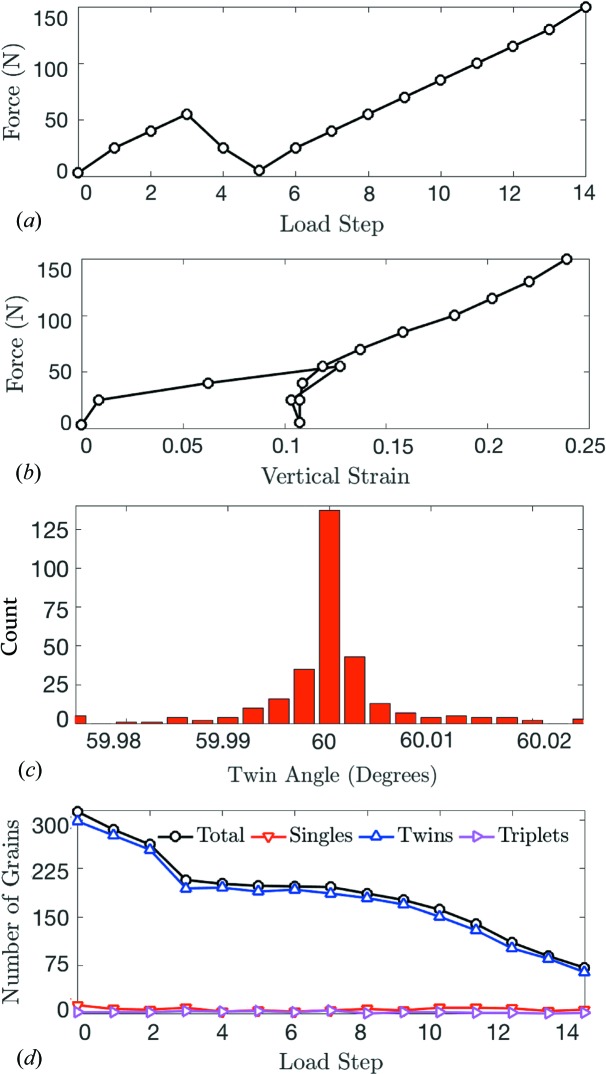
(*a*) Load-cell force as a function of load step. (*b*) Load-cell force as a function of sample vertical strain. (*c*) Distribution of angles between individual grains constituting a twinned grain. (*d*) Number of total, single, twinned and triplet grains found through *HEXRD* and grain tracking.

The granular sample was subjected to the uniaxial load path shown in Fig. 2 ▸(*a*), with each data point representing the force (as measured by the load cell) at which the sample strain was held constant while transmission radiography and diffraction measurements were made. In order to compact the initially loose sample and investigate its response to load reversal, the sample was loaded to approximately 55 N, unloaded to 5 N and reloaded to 150 N. Fig. 2 ▸(*b*) shows the response in force *versus* vertical strain space. The average macroscopic vertical strain was measured by ∊_v_ = (*h* − *h*_0_)/*h*_0_, where *h*_0_ is the sample height, manually identified as the distance between the bottom platen and the loading piston in transmission radiographs. The sample exhibited significant vertical strain with a small increase in force between steps 0 and 3. In §3.3, we show that the sample response during these steps features significant grain rotations that allowed the sample to accommodate a large strain without developing large stresses. After unloading between steps 3 and 5, the sample exhibited a steeper force–strain response because sample confinement prevented further grain rotations. Sample compaction after step 8 was probably accommodated by significant grain fracture, as illustrated in a later discussion of grain rotations in §3.3.

### 2.2. 3DXRD analysis   

The open-source software *HEXRD* (Boyce & Bernier, 2013 ▸) was used to perform analysis of the diffraction images. The analysis was performed by first providing the crystal system, the approximate crystal lattice parameters and parameters describing the instrument geometry to *HEXRD*. These parameters constrain the location used to search for diffraction peaks on the far-field area detector. Initial far-field instrument parameters including the sample-to-detector distance, intercept of the X-ray beam with the detector panel and detector tilts were found by optimizing the instrument parameters such that the predicted Debye-ring positions from a CeO_2_ standard best matched the measured data. The instrument parameters were then further refined (including the tilt of the detector about the *x* axis and the tilt of the rotation axis towards the X-ray beam) using a single-crystal ruby standard by refining the positions of predicted diffraction peaks with measured diffraction peak positions. Throughout this calibration and subsequent analysis, distortion correction terms from Lee *et al.* (2008 ▸) were used to correct the spatial distortion of the detector, which is typically done for the GE 41-RT+, as discussed by Bernier *et al.* (2011 ▸) and Borbely *et al.* (2014 ▸). In the case of the α-quartz used in this study, the crystal structure was trigonal, the space group was 152 and the initial lattice parameters, which served as a reference state, were *a* = 4.913 Å and *c* = 5.405 Å. Diffraction peaks which appeared at Bragg angles consistent with the given lattice parameters (with a small tolerance to provide for the existence of strain) and above a background threshold (20 a.u.) were isolated from the diffracted intensity generated by the aluminium tube. The isolated peaks were associated with a grain if the number of peaks for a given grain was 90% or more of the expected number for the lattice planes (for the given angular range of 280°) whose rings fell within the extent of the detector. The 90% retention (or completeness) threshold is the value recommended by the *HEXRD* developers (Bernier *et al.*, 2011 ▸).

Using the diffraction peak data for a single grain, its center of mass position, elastic strain, ∊_*ij*_, and crystal orientation were optimized so that the predicted peak positions (using previously defined instrument parameters) best match the experimental data (Bernier *et al.*, 2011 ▸). The orientation of the lattice (and subsequent rotation of the lattice) affects the azimuthal position of peaks around Debye rings on the detector. Lattice strains shift peaks radially on the detector, while the precession of grains, due to the center of mass being off the sample rotation axis, comparably perturb the positions of diffraction peaks. Crystal-lattice orientation was determined in a manner that permits evaluation of the orientation of any family of crystal planes or axes (*e.g.* the *c* axis, the *r* axis and its symmetric equivalents, *etc*.) with an absolute error of 0.05°, as reported by Bernier *et al.* (2011 ▸) and Oddershede *et al.* (2010 ▸). The distribution of relative crystal-lattice orientation for crystals comprising twinned grains (discussed later in this section) confirms 0.05° to be a reasonable estimate for the absolute error. Changes in lattice orientation from one load step to another were used to determine the rotation of grains. Centers of mass and elastic strain measurements have been previously found to have absolute errors of 10 µm and 10^−4^, respectively, using similar experimental geometries (Bernier *et al.*, 2011 ▸; Oddershede *et al.*, 2010 ▸). We confirm at the end of this section that the average absolute error in each grain’s strain component in this experiment was approximately 10^−4^.

Using the analysis described above, a total of 614 unique grains were found in step 0, with a decreasing number found in subsequent steps. At load step 14, only 365 unique grains were found. This decrease may be attributed to widespread grain comminution, which results in decreased peak intensities that fall below threshold levels and the merging of spots on the area detector into more continuous Debye–Scherrer rings. The grain-average Cauchy stress tensor (σ_*ij*_) was calculated for each grain using the anisotropic Hooke law, σ_*ij*_ = *C_ijkl_*∊_*kl*_, where *C_ijkl_* is the fourth-order elastic stiffness tensor of cultured monocrystal α-quartz from the article by Heyliger *et al.* (2003 ▸). This stress–strain relationship was applied in the crystal reference frame by transforming the stiffness tensor into the crystal frame and then transforming the resulting stress back into the sample frame. The final stress tensor for each grain in the sample reference frame was used for all analyses.

Analysis of the spatial distribution of grains revealed that the centers of mass for many grains were located within 15 µm of other grains. Because 15 µm is significantly less than the separation expected from the grain sizes retained in sieving, we further investigated the relative crystal orientation of nearby grains. We found that grains located within 15 µm of one another were relatively oriented by a nearly exact 60° rotation about the *c* axis ([0001]) of each grain. This rotational relationship suggested that the grains were actually Dauphiné twins. Dauphiné twins are penetration-type twins related to one another by a 60° rotation about the *c* axis. Because the hydrothermally grown single-crystal α-quartz blocks procured from Sawyer Technical Materials (LLC) were declared twin free by the manufacturer, it is likely that twinning occurred as a result of the high stresses generated during ball milling. Mechanical twinning has been observed in shock and impact experiments on quartz and is thought to initiate at stresses as low as 50–100 MPa (Westbrook, 1958 ▸; Laughner *et al.*, 1979 ▸; Wenk *et al.*, 2011 ▸). The stresses experienced by a plastically deforming copper target during ball milling with a steel milling ball can be as high as 600 MPa (Basset *et al.*, 1994 ▸), significantly higher than the 50–100 MPa threshold required to initiate twinning. Recent work suggests that using smaller media in the future may reduce the energy imparted to the milled material and thus reduce the development of Dauphiné twins (Herbold *et al.*, 2011 ▸).

We merged stresses of orientations corresponding to twin-related volumes if their centers of mass were within 15 µm and their orientations were within 3° of a 60° rotation about their *c* axis. However, the merging was fairly insensitive to the center-of-mass threshold: for load step 0, increasing 15 µm to 90 µm did not change the resulting number of merged grains, and most merged grains were those separated by no more than 15 µm and a 60 
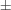
 0.05° rotation about the *c* axis. After merging, a total of 13 grains with one orientation, 298 grains with two twin-related orientations and two grains with three twin-related orientations were identified in step 0. The total number of these 313 grains matched to grains found in the XRCT image at load step 0 was 305 (97.4%). Fig. 2 ▸(*c*) shows the angle between individual crystals in the 298 twinned grains in step 0, illustrating the nearly exact 60° rotation of individual crystals making up a twin.

The stress tensors for grains with two or three twin-related orientations were found by averaging the stress tensors belonging to the two or three corresponding volumes in the sample frame. Prior to averaging, the stress tensors of twin-related volumes varied by an average of 27 and 33%, depending on load step. This percentage was calculated by 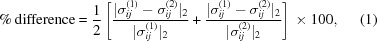
where σ_*ij*_^(1)^ and σ_*ij*_^(2)^ are the stress tensors in each of the individual twin crystals and 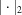 is the Frobenius norm. To justify the averaging of these different stress tensors, we calculated the relative volumes of individual crystals, *V*_r_, by comparing relative diffraction peak intensities from different orientations (diffraction intensity is proportional to crystal volume). The mean difference in relative volumes of twin orientations was less than 11% for all steps except step 10, for which it was approximately 23%. This percentage was calculated for each grain as 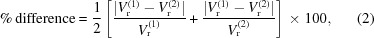
where *V*_r_^(1)^ and *V*_r_^(2)^ are the relative volumes of each of the individual twin crystals. This small difference implies a similar volume for the twin-related orientations in a grain, suggesting that an unweighted average is a close approximation to the grain-average stress. We note that further analysis of the stress differences in these individual orientations may yield interesting results, but is beyond the scope of the current paper. We also note that some grains found with a single orientation may also contain twins, since it is feasible that a twinned volume of crystal may not have been of sufficient magnitude to pass the 90% *HEXRD* retention threshold.

Grains were tracked in load steps 0 through 14 by identifying the grain in the prior load step with the nearest center-of-mass position (within 100 µm) that had rotated no more than 30° between steps. Although this criterion was developed heuristically, its accuracy is partially confirmed by the results of incremental rotation analysis provided in §3.3.[Sec sec3.3] The incremental rotations of grains between many load steps are found to be very small; most grains rotate less than 1° between steps for steps 4 through 7 and less than 5° between steps for 7 through 12. Because these rotations were computed by comparing the orientation of the grains’ crystal planes, such small rotations would be very unlikely if grains were incorrectly tracked. In fact, we have verified that no more than one grain per load step both falls within 100 µm of a neighboring grain and is oriented >60° 
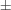
 0.05° but <5° from that neighbor.

The total number of successfully tracked grains for all load steps is shown in Fig. 2 ▸(*d*). We partially attribute the monotonic decrease in the number of successfully tracked grains to comminution. The total number of merged grains found using 3DXRD in all load steps decreases nearly monotonically from 313 in step 0 to 205 in step 14, while the number of tracked grains decreases from 313 in step 0 to 75 in step 14. The more significant reduction in the number of tracked grains suggests that some of the grains found in the 3DXRD analysis in later load steps are fragments that no longer satisfy the center-of-mass position and rotation criteria used for grain tracking. It is unlikely that intact grains would rotate or translate more than these thresholds at later steps when the sample is experiencing significant compression, as described earlier.

The stresses experienced by many grains during the experiment were greater than the 50 MPa necessary to induce twinning in dynamic experiments (see discussion in §3.2) (Westbrook, 1958 ▸; Laughner *et al.*, 1979 ▸; Wenk *et al.*, 2011 ▸). Furthermore, local grain stresses near contact points may be significantly larger than those reflected by the average stress tensor values calculated from 3DXRD. Separate research has demonstrated stress-induced twinning in α-quartz under a uni-axial load, typically at higher stresses (*e.g.* >300 MPa) than in the dynamic case (Markgraaff, 1986 ▸). We observed that between two and six grains per load step developed additional twin-related orientations (*e.g.* zero to two twin-related orientations or two to three twin-related orientations), with the exception of load step 2 for which we observed zero grains to develop additional twin-related orientations. The mean Frobenius norm of stress tensors in grains developing additional twin-related orientations was greater than 50 MPa for all load steps 8–14 (except for step 9, for which the mean was 39.4 MPa). However, the mean Frobenius norm of stress tensors for these grains was only significantly higher than that of grains that did not develop additional twin-related orientations for steps 8, 10 and 12. The Welch *t*-test (Welch, 1947 ▸), a test of significance for two populations with different standard deviations, was used to test statistical significance. The mean Frobenius norm of stress tensors in grains developing additional twin-related orientations was 74.7, 76.6 and 104.2 MPa for steps 8, 10 and 12, respectively, significantly higher than for grains not developing additional twin-related orientations at >90% confidence level [*t* = 8.0 with degrees of freedom (d.o.f.) = 1.25, *t* = 3.2 with d.o.f. = 3.5, and *t* = 2.6 with d.o.f. = 2.2, respectively].

**Figure 3:**
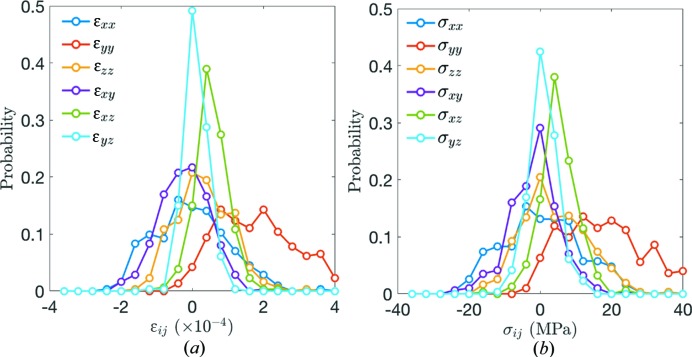
(*a*) Distribution of strain tensor components for all grains at load step 0. The standard deviations of the distributions provide approximate absolute errors. (*b*) Distribution of stress tensor components for all grains at load step 0. The standard deviations of the distributions provide approximate absolute errors.

We examined the absolute errors in grain strains and stresses as follows. First, we calculated the grain strains at load step 0 by averaging the strains in the crystals making up each grain, as was done for the stresses. The distribution of these grain strains is shown in Fig. 3 ▸(*a*) and the standard deviations are given in Table 1 ▸. These standard deviations represent approximate absolute errors for grain strain measurements; grain strains and stresses at load step 0, when the sample is uncompressed, should vanish in the absence of measurement error. As discussed earlier in this section, the errors are approximately 10^−4^ or smaller. Next, we generated 10^4^ perturbed stiffness tensors for each grain to determine the effect of uncertainties in the *C*_*ijkl*_ components and grain orientation on grain stress calculations. Perturbed stiffness tensors were generated by adding the per-component uncertainty for ordinary cultured α-quartz, given by Heyliger *et al.* (2003 ▸), multiplied by a number drawn from a standard normal distribution, to the nominal component value. We treated uncertainties as uncorrelated and thus generated a different random number for each component value (while preserving the symmetry of *C*_*ijkl*_). We further perturbed each stiffness tensor by a random rotation. Random rotations were generated from a random rotation axis and a rotation angle drawn from a normal distribution with standard deviation 0.05°. Grains strains and perturbed stiffness tensors were used to determine 10^4^ grain stress tensors per grain. The mean value of these tensors for a given grain provides an estimate of the absolute error in stress measurement. The distribution of these mean values is shown in Fig. 3 ▸(*b*) and the standard deviations of these distributions are given in Table 1 ▸. The standard deviations represent approximate absolute errors for grain stress measurements, accounting for absolute errors in strains and uncertainties in stiffness-tensor values and grain orientations.

### 2.3. XRCT analysis   

We used the ASD-POCS algorithm (Sidky & Pan, 2008 ▸) implemented in *Livermore Tomography Tools* (*LTT*) (Champley, 2016 ▸) to reconstruct three-dimensional volumes with 1.48 µm per pixel from transmission radiographs. Owing to low X-ray absorption of the sample at 51.996 keV and interference of the loading piston power cable with the edge of the rotation stage, which made an additional 5° per 180° of radiographs and 3DXRD images unusable, XRCT reconstructions featured minimal contrast between grains and voids and significant gradients in the average intensity across the image. Image binarization with a single intensity threshold for the entire image, which has been used previously, prior to watershed segmentation of grains (Hurley *et al.*, 2016 ▸; Hurley, Hall & Wright, 2017 ▸) or grain edges (Druckrey *et al.*, 2016 ▸; Cil *et al.*, 2017 ▸), was therefore inadequate, often producing large agglomerate grains. The following three-step segmentation process was therefore used: (1) the three-dimensional volume was binarized using the ‘adaptthresh’ algorithm (Bradley & Roth, 2007 ▸) in MATLAB (The Mathworks Inc., Natick, MA, USA); (2) morphological operations and watershed transforms were used to segment and label individual grains in the resulting image, similar to procedures used by Hurley and co-workers (Hurley *et al.*, 2016 ▸; Hurley, Hall & Wright, 2017 ▸); (3) grains from the 3DXRD analysis were used to further ‘split’ grains (perpendicular to lines connecting neighboring 3DXRD grains and along a plane equidistant from both grains) if two or more grains fell within the extent of each grain found in step (2). Step (3) was employed because several artificially large grains were still present in the segmentation after step (2) and the procedure in step (3) was found by inspection to provide accurate results. The XRCT image resulting from this three-step segmentation process for load step 0 is shown in Fig. 1 ▸(*b*). A total of 364 grains were identified, 305 of which (83.8%) were matched with grains from 3DXRD analysis by comparison of 3DXRD centers-of-mass with grain extents in XRCT images. The three-step segmentation process was found to yield an accurate estimate of grain shapes. The accuracy was determined both by visual inspection of individual XRCT slices and by comparing various measures of grain morphology obtained from the final images with those obtained from a segmentation of another volume of the same material, described in §2.4.

An example of a slice through the XRCT reconstruction for load step 0 is shown in Fig. 1 ▸(*c*) and the segmented three-dimensional volume at the same slice resulting from the steps described above is shown in Fig. 1 ▸(*d*). The segmentation in Fig. 1 ▸(*d*) appears to provide a reasonable approximation to the grain shapes observed in Fig. 1 ▸(*c*), as verified quantitatively in §2.4. Despite the observed accuracy of the XRCT reconstruction for load step 0 in terms of several measures of grain size and shape, many individual grains feature protrusions that make an accurate assessment of coordination number difficult. Furthermore, as grain comminution progresses, grain fragments create the further appearance of protrusions and degrade the overall image quality. We therefore used only the segmented three-dimensional volume for load step 0 in our analysis.

### 2.4. Grain morphology   

**Figure 4:**
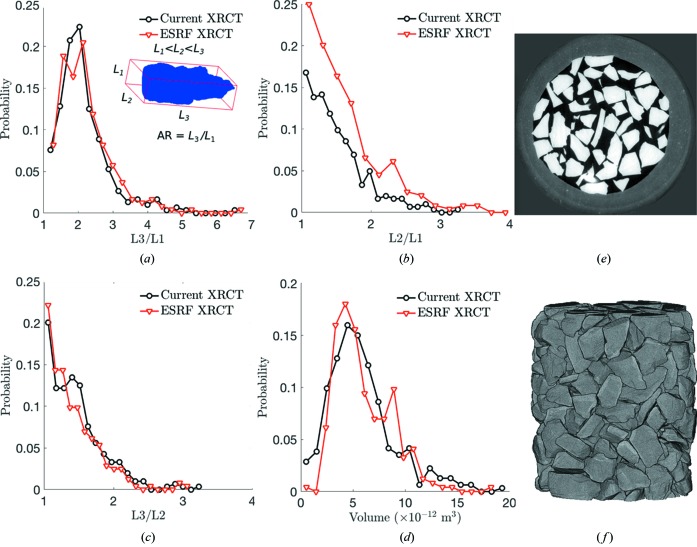
A comparison of grain morphologies from the current XRCT data and XRCT data obtained at the ESRF. Comparisons are made between (*a*) *L*_3_/*L*_1_, where *L*_3_, *L*_2_ and *L*_1_ are the bounding box lengths shown in the inset; (*b*) *L*_2_/*L*_1_; (*c*) *L*_3_/*L*_2_; (*d*) grain volumes. (*e*) A horizontal slice through an XRCT tomogram from the ESRF. (*f*) The segmented XRCT image from the ESRF.

To ensure that the three-step segmentation process described in §2.3 yielded accurate grain shapes, we compared these grain shapes with those obtained from a separate XRCT reconstruction performed on the same batch of single-crystal quartz. The separate XRCT scan was executed at the European Synchrotron Radiation Facility, beamline ID11 (ESRF, 2017 ▸). The sample consisted of 244 quartz grains poured into a rubber membrane. We obtained 1800 transmission radiographs while the sample was rotated 180° and illuminated by an X-ray box beam of 55 keV. The imaging detector stand-off distance was optimized for phase contrast and *pyHST2* was used to perform XRCT reconstructions (Mirone *et al.*, 2014 ▸). The resulting tomogram featured a pixel size of 1.54 µm. A slice of the tomogram is shown in Fig. 4 ▸(*e*) to illustrate the contrast between grains and voids. The segmentation procedures described by Hurley and co-workers (Hurley *et al.*, 2016 ▸; Hurley, Hall & Wright, 2017 ▸), using a single intensity threshold for image binarization, were then applied to the tomograms, resulting in the segmented image shown in Fig. 4 ▸(*f*).

We estimated grain volumes in XRCT images by summing the total number of pixels assigned to each grain. We estimated the orientation and principal axis lengths of each grain by applying a minimum bounding box algorithm to each grain pixel in MATLAB. The concept of this bounding box and an illustration of its maximum, intermediate and minimum axis lengths, *L*_3_, *L*_2_ and *L*_1_, respectively, is shown in the inset to Fig. 4 ▸(*a*). Fig. 4 ▸ shows a comparison between the data obtained after the three-step segmentation procedure described in §2.3 (‘Current XRC’) and the data obtained at the ESRF (‘ESRF XRCT’). A close agreement is observed between the distributions of *L*_3_/*L*_1_, *L*_2_/*L*_1_, *L*_3_/*L*_2_ and grain volumes for the current XRCT data and the data obtained at the ESRF.

We performed a further statistical comparison of each data set using Welch’s *t*-test. The *t*-test calculations for the comparisons in Figs. 4 ▸(*a*)–4(*d*) give the following results: *t* = 0.97, d.o.f. = 513 for *L*_3_/*L*_1_; *t* = −1.52, d.o.f. = 533 for *L*_3_/*L*_2_; *t* = 2.78, d.o.f. = 416 for *L*_2_/*L*_1_; *t* = 0.11, d.o.f. = 542 for grain volumes. Consulting critical values of the Student *t* distribution for a two-sided *t*-test with infinite d.o.f. (results do not significantly vary between d.o.f. = 120 and d.o.f. = ∞) reveals that the distributions for each sample compare as follows: *L*_3_/*L*_1_ are not from different distributions, even at the 70% confidence level; *L*_3_/*L*_2_ are from different distributions at the 90% but not the 95% confidence level; *L*_2_/*L*_1_ are from different distributions at the 99% confidence level; the grain volumes are not from different distributions at any reasonable confidence level. We therefore conclude that the three-step segmentation process described in §2.3 yielded accurate grain shapes in terms of grain aspect ratios *L*_3_/*L*_1_ and *L*_3_/*L*_2_ and grain volumes. These are the primary quantities used in the subsequent analysis of sample porosity, grain stresses, grain orientations and grain rotations.

## 3. Characterization of kinematics, stresses and rotations   

### 3.1. Kinematics   

**Figure 5:**
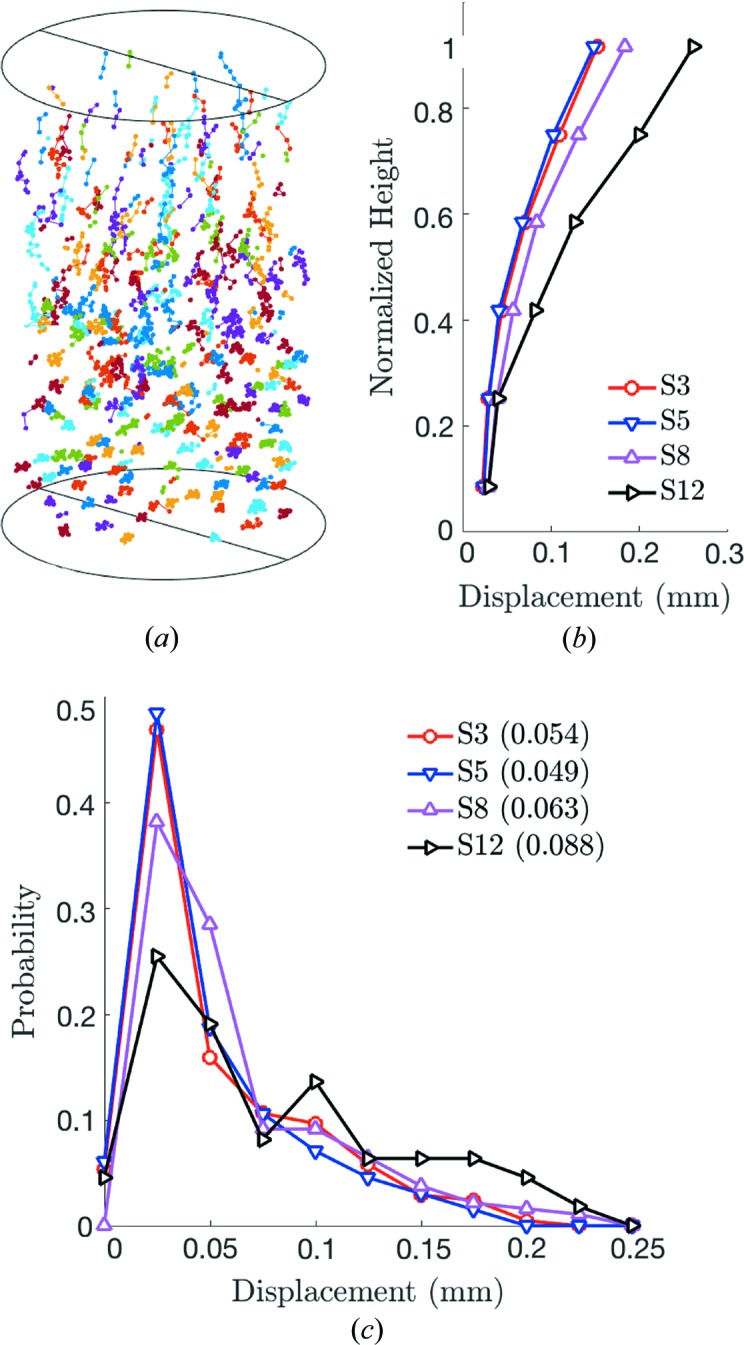
(*a*) Kinematics of grains across all load steps for which they were successfully tracked. (*b*) Average grain displacement as a function of normalized height, binned into six bins that evenly divide the sample height at the corresponding load step. S3 represents load step 3 *etc*. (*c*) Probability distribution of grain displacements at steps 3, 5, 8 and 12, with mean displacements (in mm) shown in parentheses.

The kinematics of all grains through load steps for which they were successfully tracked are shown in Fig. 5 ▸(*a*). Grains at the top of the sample experienced the most total displacement, as confirmed by the plot in Fig. 5 ▸(*b*), which shows the average displacement of all grains as a function of normalized height, (*h−z*)/*h*, where *h* is the current sample height and *z* is the *z* coordinate of the grain center of mass. The average dis­placement *versus* normalized-height curves were constructed by first binning each grain into one of six bins that evenly divide the height of the sample at the corresponding load step. The larger values of grain displacement at the top of the sample between load steps 0 (3 N) and 3 (55 N) probably occurred because the top of the sample was in a loose packing state after the grains had been poured into the cylinder. However, measurements of grain displacements at the top of the sample may be less accurate than those at the bottom; the number of particles located in bins near the top of the sample is significantly lower than the number of particles located in bins near the bottom of the sample, as shown in Table 2 ▸.

### 

Upon unloading of the sample between steps 3 and 5, the grains experienced very little rebound in height, as shown by the close alignment of curves for load steps 3 and 5 in Fig. 5 ▸(*b*) and the small leftward shift of the probability distribution of total displacements shown in Fig. 5 ▸(*c*).

### 

During reloading of the sample to 55 N at load step 8, strain ratcheting can be observed, with larger displacements at all normalized heights in Fig. 5 ▸(*b*) and a higher average displace­ment in Fig. 5 ▸(*c*), despite a similar macroscopic level of force in Fig. 5 ▸(*b*). This result is qualitatively similar to the grain kinematics and strain ratcheting observed in a separate study of spherical granular media during cyclic uniaxial compaction (Hurley, Hall & Wright, 2017 ▸). Upon further compression, the average displacements continue to increase, as shown in Figs. 5 ▸(*b*) and 5(*c*). The increase in average displacements may be the result of comminution and rearrangements. However, the significant reduction in the number of successfully tracked grains (see Fig. 2 ▸ *d*) suggests that widespread comminution may have been an important factor in the steady rise in average grain displacements with load step.

### 3.2. Stresses   

**Figure 6:**
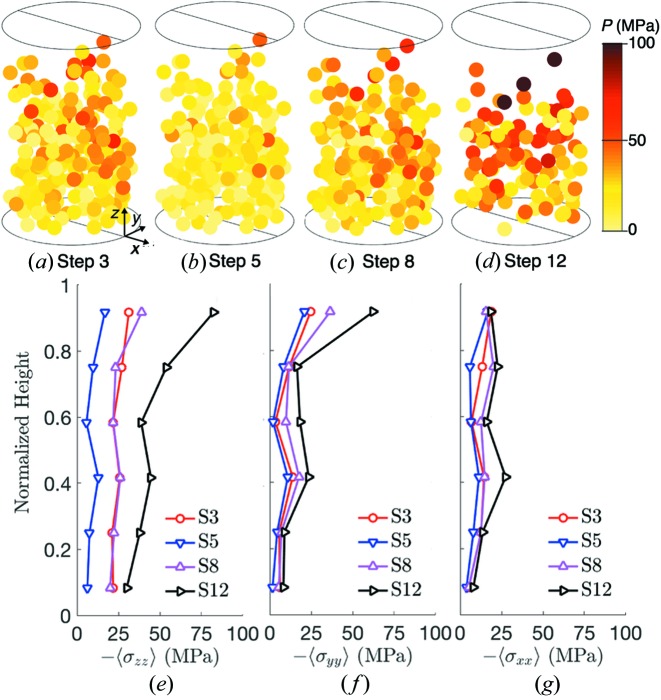
Grain pressures for grains successfully tracked and passing the *HEXRD* retention threshold at steps 3 (*a*), 5 (*b*), 8 (*c*) and 12 (*d*). Average stress as a function of normalized height for steps 3, 5, 8 and 12, for 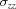 (*e*), 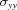 (*f*) and 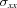 (*g*).

**Figure 7:**
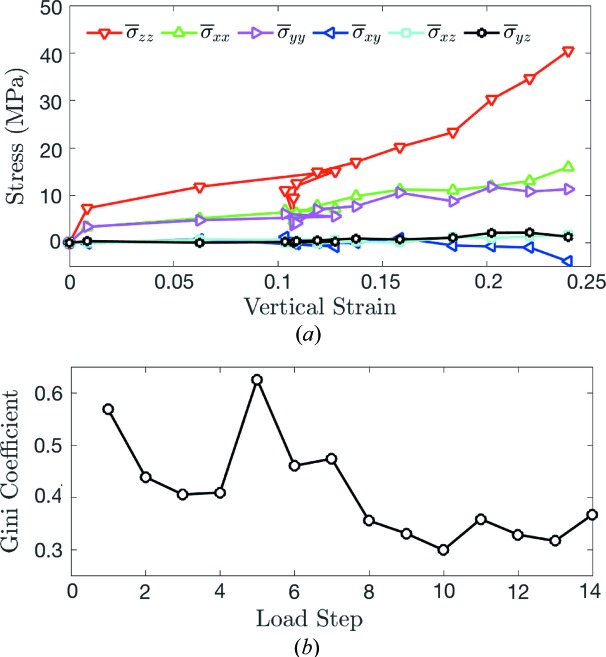
(*a*) The average stress components in each grain as a function of vertical sample strain. (*b*) The Gini coefficient of pressure in each grain as a function of load step.

The pressure (*P* = −σ_*ii*/3_) in successfully tracked grains is shown for load steps 3, 5, 8 and 12 in Figs. 6 ▸(*a*)–6(*d*), where it is rendered on circles with centers of mass corresponding to the centers of mass found during 3DXRD analysis. Fig. 6 ▸(*b*) suggests that there was a significant elastic stress release upon unloading between steps 3 and 5. This stress release is also evident in the plot of average vertical stress, σ_*zz*_, shown in Fig. 6 ▸(*e*), which is averaged over grains whose centroids fall within six equally sized bins partitioning the sample height. We note that the absolute error in the grain stress measurements is between 3.91 and 12.1 MPa, as discussed in §2.2. Those values are an upper bound on the uncertainties in values plotted in Figs. 6 ▸(*e*)–6(*g*). The actual uncertainties are are smaller: they involve averaging uncorrelated errors for each grain over all grains in a given layer. Although much of the elastic energy was released, the minimal recovery of macroscopic strain suggests that some of the energy put into the sample between load steps 0 and 5 must have been consumed by irreversible processes such as grain sliding and rearrangement. Some of the energy still appears to be stored as elastic strain energy at load step 5, reflected by the nonzero diagonal stress components in Fig. 7 ▸(*a*). Because the load-cell force returns to close to zero at load step 5, this stored elastic strain energy is likely to be induced by arching of force chains across the lateral walls of the sample (*i.e.* the aluminium cylinder).

Upon reloading of the sample from 5 N in load step 5 to 55 N in load step 8, grain pressures and the vertical distribution of stresses reached levels similar to those achieved at 55 N in load step 3, as shown in Figs. 6 ▸(*a*), 6(*c*), 6(*e*) and 6(*f*). This result is qualitatively similar to the results of a study on spherical granular materials undergoing cyclic loading (Hurley, Hall & Wright, 2017 ▸) that showed a recovery of elastic grain stresses across load cycles. Finally, upon further loading to 120 N at load step 12, we observed a stiffening response, consistent with the increase in slope of Figs. 2 ▸(*a*) and 2(*b*). This stiffening may have occurred because of widespread grain comminution, which was also observed and accompanied by a stiffening response in spherical grains mixed with ductile copper at similar strain levels by Hurley *et al.* (2018 ▸). We hypothesize that comminution was responsible for stiffening in the current experiment because either grain rearrangement, comminution or both are needed to produce force chain structures that are better suited to support applied loads. However, significant grain rearrangement alone is unlikely after load step 8, when the sample porosity reached 0.44, as judged by the change in sample height and the grain volumes computed from the XRCT image at load step 0. Grain comminution enhances grain rearrangement by creating additional d.o.f. and void space into which intact grains can move.

Fig. 7 ▸(*a*) shows the evolution of volume-averaged grain stress components, 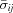, across load steps 0 through 14, computed by 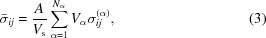
where *V*_s_ is the sample volume, *V*_α_ is the volume of grain α as determined from the segmented XRCT reconstruction of load step 0, σ_*ij*_^(α)^ is the stress tensor in grain α and *A* = 364/*N* is the ratio of tracked grains from the 3DXRD data at a particular load step to total grains observed in the XRCT segmentation of load step 0. The scale factor *A* accounts for the change in 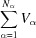 as the experiment progresses. *A* should remain constant if all grains and grain fragments were successfully tracked. The vertical stress, 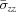, evolves in a similar manner to the load-cell force observed in Fig. 2 ▸(*b*). Fig. 7 ▸(*b*) shows the evolution of the Gini coefficient of grain pressures as a function of the load step, computed by 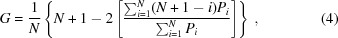
where *P_i_* is an ordered array of grain pressures such that *P_i_* ≤ *P*_*i*+1_ for *i* from 1 to *N*, the total number of tracked grains. This Gini coefficient quantifies the heterogeneity of grain pressures throughout the sample, with a value of 1 representing a completely heterogenous population in which one grain carries all the pressure of the system and a value of 0 representing complete homogeneity in which all grains carry an equal pressure (Hurley *et al.*, 2016 ▸). The Gini coefficient in Fig. 7 ▸(*b*) approximately follows the opposite trend of the load-cell force in Fig. 2 ▸(*a*), implying that stresses become more homogeneous when the granular sample is under increasing load. Although some grains are not tracked in our analysis, potentially biasing results, the increase in pressure homogeneity with load is qualitatively similar to the increase in the interparticle force homogeneity observed in separate studies of the compaction of spherical granular materials (Hurley *et al.*, 2016 ▸; Hurley, Hall & Wright, 2017 ▸; Makse *et al.*, 2000 ▸).

### 3.3. Rotations   

The orientation of each crystal was determined in terms of an angle axis parameterization by *HEXRD*, as described in §2.2. An internal function in *HEXRD* was used to calculate the rotation of grains from changes in lattice orientation in subsequent load steps while accounting for possible ambiguity due to crystal symmetries. In addition to permitting the following analysis, calculating grain rotations provided a partial confirmation of the accuracy of grain tracking, as described in §2.2.

**Figure 8:**
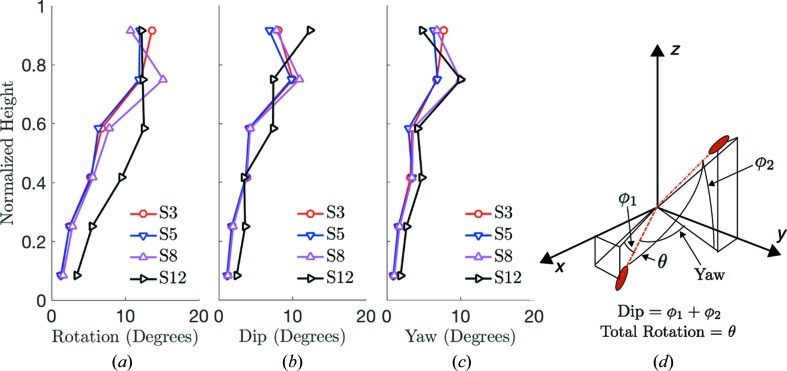
(*a*) Average total grain rotation, from load step 0, as a function of normalized height for steps 3, 5, 8 and 12. (*b*) Average total dip of each grain from load step 0 as a function of normalized height. (*c*) Average total yaw of each grain from load step 0 as a function of normalized height. (*d*) A figure illustrating the meaning of total rotation (θ), dip (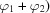 and yaw, by using an example of angles between two orientations of an elongated ellipsoid. The orientation of the ellipsoid is also illustrated by dashed lines that connect its principal axis to the origin.

Fig. 8 ▸ illustrates the average total rotation, dip and yaw of each grain, relative to its orientation in load step 0, as a function of normalized height for steps 3, 5, 8 and 12. The dip is the change in vertical orientation of a grain, independent of its rotation in the *x* *y* plane. The yaw is the rotation of a grain in the *x* *y* plane, independent of its change in vertical orientation. The concept of these rotation angles is shown in Fig. 8 ▸(*d*). We note that the relative error in calculating the orientation of an individual grain using 3DXRD is approximately 0.05° (Oddershede *et al.*, 2010 ▸). A significant amount of total rotation, dip and yaw occurred up to load step 3, at which point the sample was compressed by 55 N of force. During unloading to 5 N between load steps 3 and 5, grain rotations in all directions were minimal throughout the sample. Consistent with prior analysis of the reversal of displacements and stresses, this finding suggests that most of the energy put into the sample between load steps 0 and 5 was dissipated by grain sliding (resulting from rotations and rearrangements) or stored in elastic strain energy caused by horizontal force-chain arching (see §3.2).

Upon reloading of the sample to load step 8, the average total rotation and yaw increased toward the highest points in the sample, whereas the average dip remained nearly constant at all heights. Finally, at load step 12, increases in all rotations (total, dip and yaw) were observed at nearly all heights. At the highest points in the sample, the total rotation remained nearly constant between load steps 8 and 12, while the average dip increased and the average yaw decreased. In addition, the average dip was slightly higher than the average yaw at most points in the sample throughout the experiment, suggesting a slightly higher likelihood of grain rotation relative to the loading axis rather than around the loading axis. This tendency is explored further in subsequent analysis.

**Figure 9:**
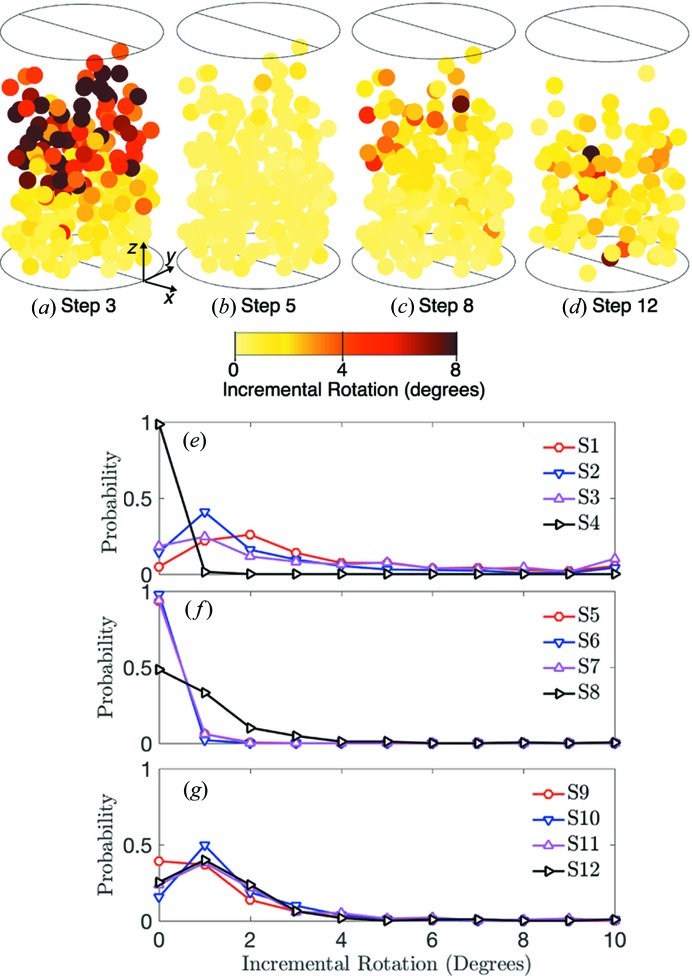
Incremental total rotation of grain orientations for load steps 3 (*a*), 5 (*b*), 8 (*c*) and 12 (*d*). Probability distributions of incremental total rotations for load steps 1–4 (*e*), 5–8 (*f*) and 9–12 (*g*).

**Figure 10:**
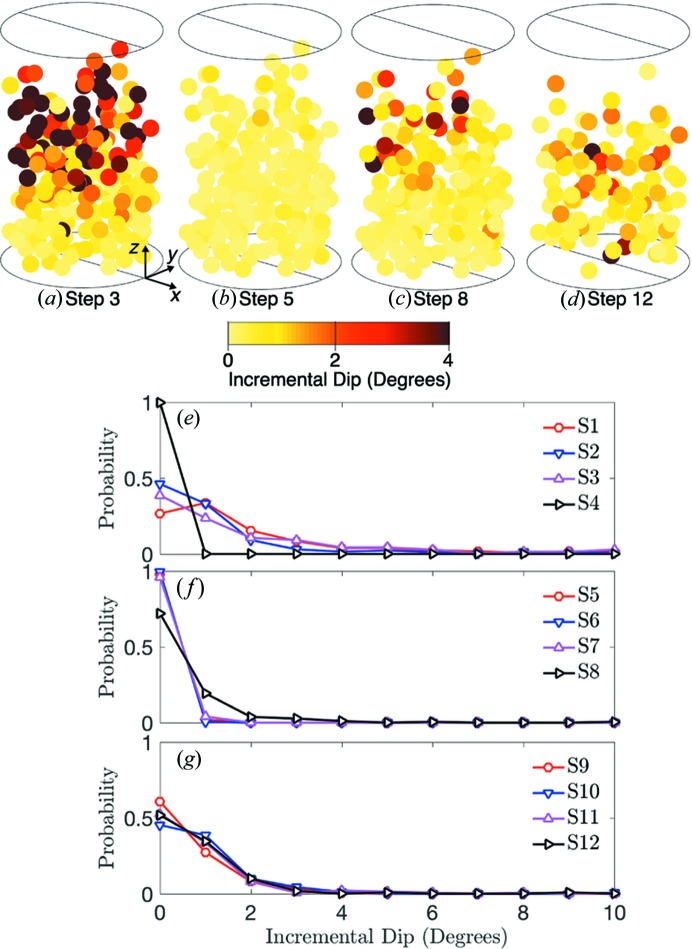
Incremental dip of grain orientations for load steps 3 (*a*), 5 (*b*), 8 (*c*) and 12 (*d*). Probability distributions of incremental dip for load steps 1–4 (*e*), 5–8 (*f*) and 9–12 (*g*).

**Figure 11:**
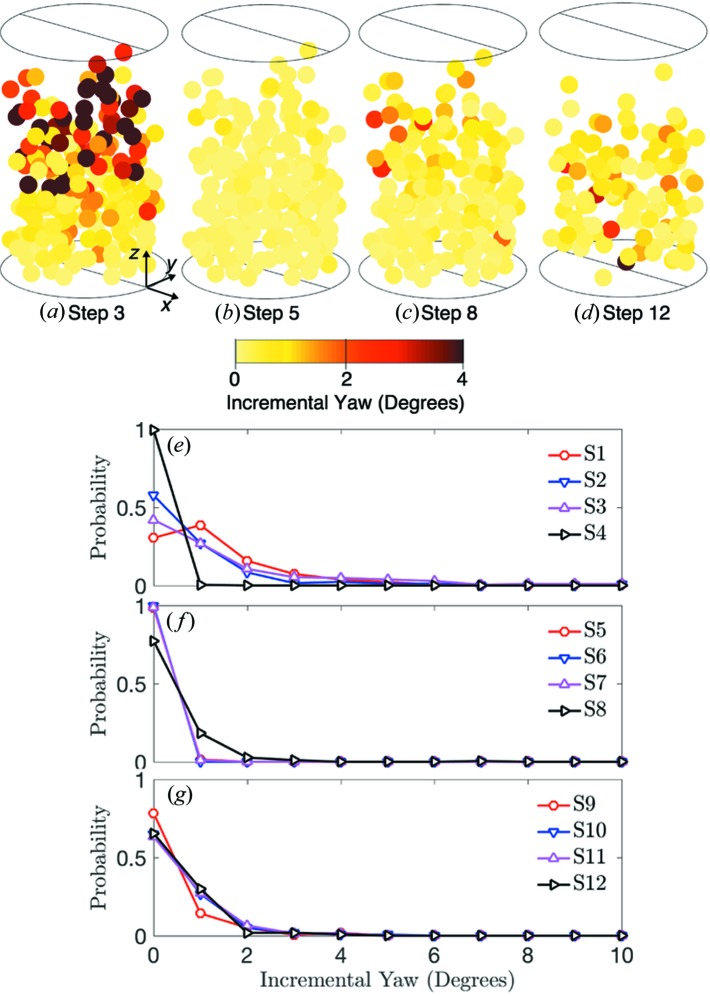
Incremental yaw of grain orientations for load steps 3 (*a*), 5 (*b*), 8 (*c*) and 12 (*d*). Probability distributions of incremental yaw for load steps 1–4 (*e*), 5–8 (*f*) and 9–12 (*g*).

To gain deeper insight into grain rotations, we plot the incremental total rotation, incremental dip and incremental yaw for load steps 3, 5, 8 and 12 and the probability distribution rotations for steps 1–12 in Figs. 9, 10 and 11, respectively. We note that the probability distributions may not be representative of all grains and grain fragments at each load step because they are calculated only for tracked grains. Incremental rotations were found by comparing the orientation of grains between load steps. Significant incremental rotation, dip and yaw can be observed in load steps 1–3 in Figs. 9(*e*), 10(*e*) and 11(*e*), respectively. These incremental rotations occurred primarily at the top of the sample as shown in Figs. 9(*a*), 10(*a*) and 11(*a*). We identify these rotations as those which accommodate the initial compaction of the sample from a loose to a dense state. Upon unloading to 5 N and reloading to 55 N in steps 4–8, very little rotation, dip and yaw can be observed in Figs. 9, 10 and 11.

When the sample was loaded above 55 N in steps 8–14, we observed additional grain rotations, as shown in Figs. 9 ▸(*c*), 9(*d*) and 9(*g*), Figs. 10 ▸(*c*), 10(*d*) and 10(*g*), and Figs. 11 ▸(*c*), 11(*d*) and 11(*g*). Because the sample was highly confined at and beyond load step 8, we hypothesized that these rotations were facilitated by nearby grain comminution. We tested this hypothesis by computing the correlation coefficient between the distance from a grain to the nearest grain whose tracking was lost in the current step and the incremental rotation of the grain. We found a negative correlation coefficient for all load steps except step 7, and an average correlation coefficient of −0.1675 for steps 8 through 14. This implies that grains closer to those whose tracking was lost experienced slightly larger incremental rotations than those farther away. Although this provides some evidence that grain comminution is aiding in further rotations after load step 7, this coupling between fracture and rotational deformation mechanisms deserves further study.

### 3.4. Correlations between rotations and other responses   

**Figure 12:**
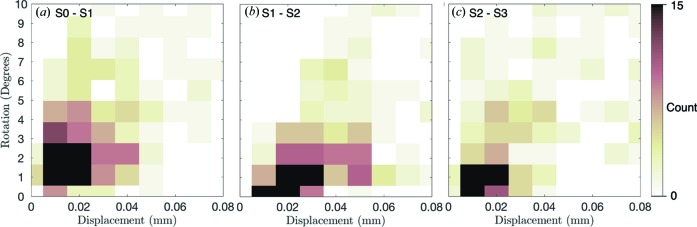
Density of incremental displacements and incremental rotations of grains between load steps 0 and 1 (*a*), 1 and 2 (*b*), and 2 and 3 (*c*)

We investigated correlations between individual grain rotations and grain displacements, initial angularity and stress responses. Fig. 12 ▸ shows a density plot of the incremental displacement *versus* the incremental rotation angle for all grains between load steps 0 and 1 (*a*), steps 1 and 2 (*b*), and steps 2 and 3 (*c*). There were a variety of displacement and rotation responses in the first three steps of compaction, with some grains rotating as much as 9° while translating only 0.02 mm (Fig. 12 ▸ *a*), and other grains translating as much as 0.07 mm with only 1° of rotation (Fig. 12 ▸ *b*). There is, however, an apparent positive correlation between incremental grain displacement and rotation in all figures. A similar positive correlation was observed between incremental grain displacements and both dips and yaws, but figures for these are not shown for brevity.

**Figure 13:**
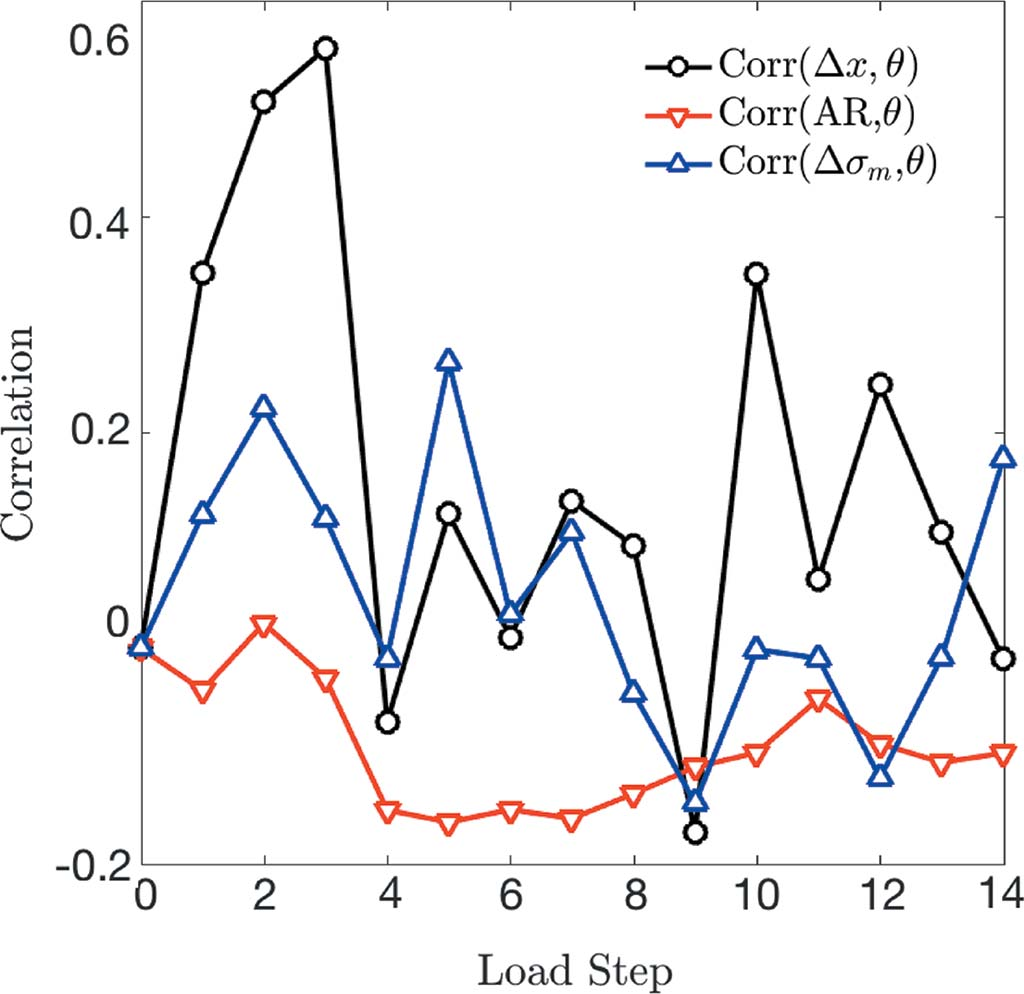
Correlation coefficients between incremental rotation, θ, and incremental displacements, 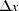, initial aspect ratio, AR = *L*_3_/*L*_1_, and incremental change in von Mises stress, 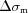.

**Figure 14:**
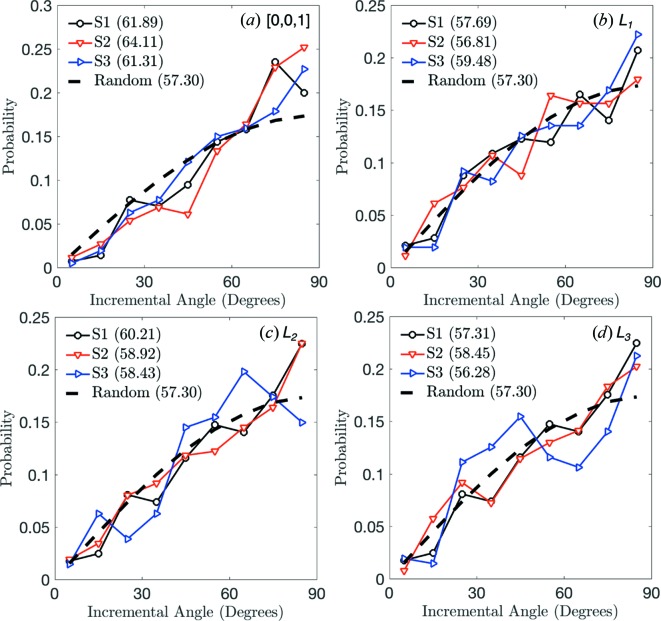
Probability distribution of the angle between the incremental rotation axes of the grains and the *z* axis ([001]) (*a*), the *L*_1_-axis of each grain bounding box (*b*), the *L*_2_ axis (*c*) and the *L*_3_ axis (*d*).

In Fig. 13 ▸(*a*), we compute the correlation coefficient between incremental displacement, Δ*x*, and incremental rotation, θ, as 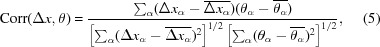
where α is a grain index and the overbar represents an average. There is a clear and increasing correlation between Δ*x* and θ, as conveyed in Fig. 13 ▸, in load steps 1 through 3, followed by a rapid decrease in correlation upon unloading in load step 4. We suspect that this correlation arises because loosely packed grains near the top of the sample are preferentially rotating about a rotation axis with a high dip angle to the *z* axis in order to accommodate compaction (*e.g.* by ‘flattening’ their vertical profile). We confirm this hypothesis in Fig. 14 ▸(*a*), in which we plot the probability distribution of the angle between the grains’ incremental rotation axes and the *z* direction, [001]. We observe the angle between each grain’s rotation axis and [001] (average of 61.89° for step 1, 64.11° for step 2 and 61.31° for step 3) to be slightly higher than expected from a random distribution of rotation axes (average of approximately 57.3°). We have verified that this result is statistically significant at the 99% confidence level according to a Student *t*-test for load steps 1 (*t* = 3.82, d.o.f. = 284), 2 (*t* = 5.35, d.o.f. = 261) and 3 (*t* = 2.86, d.o.f. = 206).

Fig. 13 ▸ also shows the correlation coefficient between each grain’s initial aspect ratio, AR = *L*_3_/*L*_1_, where *L*_3_ and *L*_1_ are the maximum and minimum bounding box side lengths for the grain, and θ. This coefficient is computed using equation (5) by replacing Δ*x*_α_ with AR_α_. Unlike with Corr(Δ*x*, θ), we observe almost no correlation between the initial aspect ratio of a grain and the amount of incremental rotation it experiences at all load steps. This is confirmed in Figs. 14 ▸(*b*)–14(*d*), in which we show the probability distributions of the dip angle between the incremental rotation axes of the grains and the bounding box axes *L*_1_, *L*_2_ and *L*_3_. We observe the dip angles to be distributed around the value expected from a random distribution of rotation axes. Student *t*-tests confirm that none of these dip angles differ from what is expected from a random distribution of rotation axes at the 90% confidence level, with the exception of the dip angles to *L*_2_ for step 1, which show a significant difference at the 98% confidence level (*t* = 2.24, d.o.f. = 284). A possible explanation for the minimal influence of each grain’s initial AR on its rotation is given in the next subsection.

Finally, in Fig. 13 ▸ we also show the correlation between θ and each grain’s incremental change in von Mises stress, Δσ_m_. The von Mises stress is given by 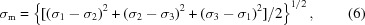
where σ_1_, σ_2_ and σ_3_ are the principal stresses. Von Mises stresses were chosen as a point of comparison because they reflect the amount of distortional strain energy in each grain. This distortional energy acts to effect a change in shape or orientation of a grain by shearing. As with AR, we observe almost no correlation between incremental changes in σ_m_ of a grain and its incremental rotations. This finding may reflect the significance of sample confinement in that grains exhibiting large rotations do not undergo significant stress drops as may be expected in a loose material. We have also confirmed a lack of correlation between the incremental changes in other stress measures (*e.g.* pressure and individual stress tensor components) and rotations.

### 3.5. Evolution of grain orientations   

**Figure 15:**
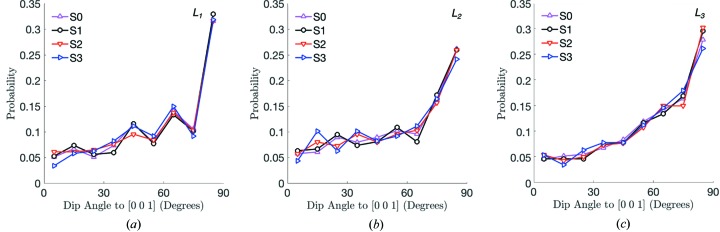
Dip angle between the *z* axis, [001], and each grain’s bounding box axes, 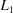 (*a*), 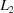 (*b*) and 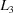 (*c*).

It is important to understand why the initial AR of a grain is not related to its rotations. To this end, any preferential alignment of the grains during sample preparation is a possible explanation. To study preferential grain alignment during sample preparation, we computed the dip angle (different from the dip rotation described earlier) between the *L*_1_, *L*_2_ and *L*_3_ axes of each grain and the *z* axis, [001]. Fig. 15 ▸ shows the dip angle for load steps 0, 1, 2 and 3. All grains demonstrate a strong preference to orient one of their bounding box axes orthogonally to the *z* axis of the sample at load step 0 before significant force is applied to the sample. However, grains do not tend to significantly reorient themselves to minimize their height after load step 0, suggesting that they are restricted to the orientation and approximate potential energy level that they take during sample preparation. We contend that this kinematic restriction is responsible for the negligble correlation between AR and θ. A similar phenomenon may occur in other geometries with similar levels of lateral confinement, implying that materials cannot always minimize their potential energy after initial compaction. This important point should be further investigated with additional experiments and simulations, as it may have implications for the fundamental assumptions made in continuum modeling of granular materials.

## 4. Summary and conclusions   

We employed 3DXRD to characterize intra-grain crystal structure, kinematics, stresses and rotations in granular quartz during uniaxial compaction. This work marked a departure from previous studies employing spherical single-crystal grains (Hurley, Hall & Wright, 2017 ▸; Hurley, Lind *et al.* 2017 ▸; Hurley *et al.* 2018 ▸) and instead used angular quartz grains containing Dauphíne twins. By doing so, the study conveys how 3DXRD can be used to study granular mechanics in angular particles and represents a step toward studying the types of angular powders encountered frequently in nature and industry. Some of this work has already been performed in other studies (*e.g.* Cil *et al.*, 2017 ▸; Imseeh & Alshibli, 2018 ▸). By analyzing correlations between various mechanical responses and comparing the results of the present study with prior studies employing spherical grains, we reach the following conclusions:

(1) The angular granular materials exhibited strain ratcheting, stress recovery and stress heterogeneity that was qualitatively similar to that observed in spherical granular materials subjected to uniaxial cyclic loading in cylindrical geometries.

(2) There was a positive correlation between grain rotations and translations at the onset of loading, but very little correlation between grain aspect ratio and rotation or a change in any measure of a grain’s incremental stress change and rotation.

(3) During initial compaction, grains rotated about an axis significantly inclined to the loading direction (with statistical significance).

(4) Angular grains may preferentially orient their bounding box axes relative to the cylinder axis in samples prepared by pouring under the influence of gravity, making the initial aspect ratio less important in determining which grains exhibit significant rotations.

(5) Grains composed of materials like quartz may experience stress-induced twinning during deformation.

Most studies to date have employed the cylindrical sample geometry used here and a relatively small number of grains (less than 1100). We believe that additional research is needed with samples containing more grains in order to draw further conclusions about the correlations between various mechanical responses. Nevertheless, the work presented here has elucidated various aspects of the mechanical response of angular grains and illustrates new applications of 3DXRD to studying crystal structure and rotations in granular materials.

## Figures and Tables

**Table 1:** Average absolute errors in ∊_*ij*_ and σ_*ij*_ for each grain, determined using the procedure described in the text

Component	∊_*ij*_ absolute error (×10^−4^)	σ_*ij*_ absolute error (MPa)
*xx*	1.01	11.0
*yy*	1.12	12.1
*zz*	0.74	8.87
*xy*	0.68	7.09
*xz*	0.46	4.95
*yz*	0.31	3.91

**Table 2:** Number of particles located in the bins centered at each normalized height in Fig. 5(*b*) for all load steps shown These numbers also hold for Figs. 6(*e*)–6(*g*) and 8(*a*)–8(*c*).

Normalized height	Step 3	Step 5	Step 8	Step 12
0.917	10	7	8	4
0.75	24	21	16	9
0.583	39	32	32	28
0.417	43	46	42	19
0.25	46	47	44	24
0.083	45	45	44	26

## References

[bb2] Alshibli, K. A., Batiste, S. N., Swanson, R. A., Sture, S., Costes, N. C. & Lankton, M. R. (2000). *Geotechnical Measurements: Lab and Field*, American Society of Civil Engineers.

[bb1] Alshibli, K., Cil, M. B., Kenesei, P. & Lienert, U. (2013). *Granular Matter*, **15**, 517–530.

[bb3] Alshibli, K. A., Sture, S., Costes, N. C., Frank, M. L., Lankton, M. R., Batiste, S. N. & Swanson, R. A. (2000). *Geotech. Test. J.* **23**, 274–299.

[bb4] Aste, T., Saadatfar, M., Sakellariou, A. & Senden, T. J. (2004). *Physica A*, **339**, 16–23.

[bb5] Aste, T., Saadatfar, M. & Senden, T. (2005). *Phys. Rev. E*, **71**, 061302. 10.1103/PhysRevE.71.061302 16089730

[bb6] Basset, D., Matteazzi, P. & Miani, F. (1994). *Mater. Sci. Eng. A*, **174**, 71–74.

[bb7] Bernier, J., Barton, N., Lienert, U. & Miller, M. (2011). *J. Strain Anal. Engineering Des.* **46**, 527–547.

[bb8] Bésuelle, P., Desrues, J. & Raynaud, S. (2000). *Int. J. Rock Mech. Min. Sciences*, **37**, 1223–1237.

[bb9] Borbely, A., Renversade, L., Kenesei, P. & Wright, J. (2014). *J. Appl. Cryst.* **47**, 1042–1053.

[bb10] Boyce, D. & Bernier, J. (2013). *heXRD: Modular, Open Source Software for the Analysis of High Energy X-ray Diffraction Data.* Technical Report, Lawrence Livermore National Laboratory, Livermore, CA, USA.

[bb11] Bradley, D. & Roth, G. (2007). *J. Graphics Tools*, **12**, 13–21.

[bb12] Champley, K. (2016). *Livermore Tomography Tools (LTT) Technical Manual.* LLNL Technical Report (under development), Lawrence Livermore National Laboratory, Livermore, CA, USA.

[bb13] Chen, K.-C. & Lan, J.-Y. (2009). *Int. J. Solids Struct.* **46**, 1554–1563.

[bb14] Cil, M. & Alshibli, K. (2014). *Géotechnique*, **64**, 351.

[bb15] Cil, M. B., Alshibli, K. A. & Kenesei, P. (2017). *J. Geotech. Geoenviron. Eng.* **143**, 04017048.

[bb16] Desrues, J., Chambon, R., Mokni, M. & Mazerolle, F. (1996). *Géotechnique*, **46**, 529–546.

[bb17] Desrues, J., Viggiani, G. & Besuelle, P. (2010). *Advances in X-ray Tomography for Geomaterials*. London: Wiley ISTE.

[bb18] Druckrey, A. M. & Alshibli, K. A. (2016). *Int. J. Numer. Anal. Methods Geomech.* **40**, 105–116.

[bb19] Druckrey, A. M., Alshibli, K. A. & Al-Raoush, R. I. (2016). *Comput. Geotechnics*, **74**, 26–35.

[bb20] ESRF (2017). *ID11 – Materials Science Beamline*, http://www.esrf.eu/UsersAndScience/Experiments/StructMaterials/ID11.

[bb21] Goddard, J., *et al.* (2007). *Powder Grains*, **1**, 129–134.

[bb22] Hall, S. A. & Wright, J. (2015). *Géotechnique Lett.* **5**, 236–242.

[bb23] Hall, S. A., Wright, J., Pirling, T., Andó, E., Hughes, D. J. & Viggiani, G. (2011). *Granular Matter*, **13**, 251–254.

[bb24] Herbold, E. B., Jordan, J. L. & Thadhani, N. (2011). *Acta Mater.* **59**, 6717–6728.

[bb25] Heyliger, P., Ledbetter, H. & Kim, S. (2003). *J. Acoust. Soc. Am.* **114**, 644–650. 10.1121/1.1593063 12942948

[bb26] Hurley, R., Hall, S., Andrade, J. & Wright, J. (2016). *Phys. Rev. Lett.* **117**, 098005. 10.1103/PhysRevLett.117.098005 27610890

[bb28] Hurley, R. C., Hall, S. A. & Wright, J. (2017). *Proc. R. Soc. London A*, **473**, 0491.

[bb27] Hurley, R., Lind, J., Pagan, D., Akin, M. & Herbold, E. (2018). *J. Mech. Phys. Solids*, **112**, 273–290.

[bb29] Hurley, R. C., Lind, J., Pagan, D. C., Homel, M. A., Akin, M. C. & Herbold, E. B. (2017). *Phys. Rev. E*, **96**, 012905. 10.1103/PhysRevE.96.012905 29347136

[bb30] Imseeh, W. H. & Alshibli, K. A. (2018). *Comput. Geotechnics*, **94**, 184–195.

[bb31] Kawamoto, R., Andò, E., Viggiani, G. & Andrade, J. E. (2016). *J. Mech. Phys. Solids*, **91**, 1–13.

[bb32] Laughner, J., Cline, T., Newnham, R. E. & Cross, L. (1979). *Phys. Chem. Miner.* **4**, 129–137.

[bb33] Lee, J. H., Aydıner, C. C., Almer, J., Bernier, J., Chapman, K. W., Chupas, P. J., Haeffner, D., Kump, K., Lee, P. L., Lienert, U., Miceli, A. & Vera, G. (2008). *J. Synchrotron Rad.* **15**, 477–488. 10.1107/S090904950801755X 18728319

[bb34] Makse, H. A., Johnson, D. L. & Schwartz, L. M. (2000). *Phys. Rev. Lett.* **84**, 4160–4163. 10.1103/PhysRevLett.84.4160 10990635

[bb35] Markgraaff, J. (1986). *Phys. Chem. Miner.* **13**, 102–112.

[bb36] Mirone, A., Brun, E., Gouillart, E., Tafforeau, P. & Kieffer, J. (2014). *Nucl. Instrum. Methods Phys. Res. B*, **324**, 41–48.

[bb37] Oddershede, J., Schmidt, S., Poulsen, H. F., Sørensen, H. O., Wright, J. & Reimers, W. (2010). *J. Appl. Cryst.* **43**, 539–549.

[bb38] Sakellariou, A., Arns, C. H., Sheppard, A. P., Sok, R. M., Averdunk, H., Limaye, A., Jones, A. C., Senden, T. J. & Knackstedt, M. A. (2007). *Mater. Today*, **10**, 44–51.

[bb39] Sidky, E. Y. & Pan, X. (2008). *Phys. Med. Biol.* **53**, 4777–4807. 10.1088/0031-9155/53/17/021 PMC2630711 18701771

[bb40] Turner, M., Knüfing, L., Arns, C., Sakellariou, A., Senden, T., Sheppard, A., Sok, R., Limaye, A., Pinczewski, W. V. & Knackstedt, M. (2004). *Physica A*, **339**, 166–172.

[bb41] Welch, B. L. (1947). *Biometrika*, **34**, 28–35. 10.1093/biomet/34.1-2.28 20287819

[bb42] Wenk, H.-R., Janssen, C., Kenkmann, T. & Dresen, G. (2011). *Tectonophysics*, **510**, 69–79.

[bb43] Westbrook, J. (1958). *J. Am. Ceram. Soc.* **41**, 433–440.

